# Prenatal folate, homocysteine and vitamin B12 levels and child brain volumes, cognitive development and psychological functioning: the Generation R Study – CORRIGENDUM

**DOI:** 10.1017/S0007114526106278

**Published:** 2026-05-14

**Authors:** Charlotte L. Ars, Ilse M. Nijs, Hanan El Marroun, Ryan Muetzel, Marcus Schmidt, Jolien Steenweg-de Graaff, Aad van der Lugt, Vincent W. Jaddoe, Albert Hofman, Eric A. Steegers, Frank C. Verhulst, Henning Tiemeier, Tonya White

In the original publication, the co-author Hanan El Marroun’s name was misspelled as ‘Hanan E. Marroun’.

This has since been updated in the published paper.
